# A comprehensive survey of government auditors’ self-efficacy and professional development for improving audit quality

**DOI:** 10.1186/s40064-016-2903-0

**Published:** 2016-08-05

**Authors:** Shue-Ching Lee, Jau-Ming Su, Sang-Bing Tsai, Tzu-Li Lu, Weiwei Dong

**Affiliations:** 1 Genomic Research Center, Academia Sinica, Taipei, 115 Taiwan; 2 Ph.D. Program of Technology Management, Chung Hua University, 707, Sec.2, WuFu Rd., Hsinchu, Hsinchu, 300 Taiwan, ROC; 3 Department of Transportation Technology and Logistics Management, Chung Hua University, 707, Sec.2, WuFu Rd., Hsinchu, 300 Taiwan, ROC; 4 Zhongshan Institute, University of Electronic Science and Technology of China, Zhongshan, 528402 China; 5 School of Economics and Management, Shanghai Maritime University, Shanghai, 201306 China; 6 Law School, Nankai University, Tianjin, 300071 China; 7 School of Business, Dalian University of Technology, Panjin, 124221 China; 8 Department of International Trade, Takming University of Science and Technology, Taipei, 114 Taiwan; 9 School of Economics and Management, Shanghai Institute of Technology, Shanghai, 201418 China

**Keywords:** Self-efficacy, Professional development, Mentoring, Knowledge sharing, Audit quality, Government auditing, Management

## Abstract

**Introduction:**

Government audit authorities supervise the implementation of government budgets and evaluate the use of administrative resources to ensure that funding is used wisely, economically, and effectively. A quality audit involves reviewing policies according to international standards and perspectives, and provides insight, predictions, and warnings to related organizations. Such practice can reflect the effectiveness of a government.

**Case description:**

Professional development and self-efficacy have strong influence upon the performance of auditors. To further understand the factors that may enhance their performance and to ultimately provide practical recommendations for the audit authorities, we have surveyed about 50 % of all the governmental auditors in Taiwan using the stratified random sampling method.

**Discussion and Evaluation:**

The result showed that any auditing experience and professionalization can positively influence the professional awareness. Also, acquired knowledge and skillset of an auditor can effectively improve ones professional judgment. We also found that professional development (including organizational culture and training opportunities) and self-efficacy (including profession and experience as well as trends and performance) may significantly impact audit quality.

**Conclusions:**

We concluded that to retain auditors, audit authorities must develop an attractive future outlook emphasizing feedback and learning within an organization. Our study provides a workable management guidelines for strengthening the professional development and self-efficacy of audit authorities in Taiwan.

## Introduction

Audit authorities supervise the implementation of government budgets and evaluate legitimate use of administrative resources to ensure funding being used wisely, economically, and effectively. In practice, besides inspecting existing financial records, government audit authorities should maximize the use of resources by ensuring that the administrative units, departments, and sections of the executive branch achieve their desired goals economically and efficiently. Since the quality of governmental auditing services not only reflects how the government functions but also influences how people view the government and its executive branches. Under the intense wave of globalization, changes are accompanied by technological innovations, economic liberalization and increasing awareness of an active citizenship, all of which in turn bring tremendous challenges to the Taiwan government in many frontlines including governmental reform, economic growth, social welfare, tax revenues, anti-corruption, insurance and pension plans. These issues are strongly influenced by international events, the government’s policies and finance regulations. The ranking of Taiwan by IMD (International Institute for Management Development an organized which ranks country’s performance based on economic performance, government efficiency, business efficiency, and infrastructure) is lower than many other Asian countries, especially in government efficiency. Thus, ensuring audit quality becomes an important approach to increase the value of resources and to stimulate economic development. Governmental audit quality plays an essential role for the effectiveness of an administration. A quality audit involves reviewing policies according to international standards and perspectives, and provides insight, predictions, and warnings to related organizations. Such practice can reflect the effectiveness of a government.

DeAngelo (1981) defined audit quality as “the market-assessed joint probability that a given auditor will both (a) discover a breach in a client’s accounting system and (b) report the breach.” A number of scholars emphasized that, for auditors, an audit quality implies that the audit is accomplished according to the methodology or guideline defined by the audit authority. As for audit authorities, an audit quality means that the audit report can withstand the challenge in court (Knechel et al. 2013). As noted by social scientists, factors that can improve audit quality include (1) intensive training (Knechel et al. 2013), (2) audit specialization and execution of error detection, procedure analysis, audit risk evaluation, and internal control deficiency discovery (Stephens 2011), (3) the knowledge and skills to make professional decisions (Knechel 2010; Bobek et al. 2012), and (4) the professionalism of the auditors (Nagy 2012). Specialization has become more important in the current auditing environment, and the auditing team characteristic has evolved into one of the crucial factors for audit quality. In today’s dynamic and demanding economic environment, professional auditors need to maintain competence and knowledge of current developments to enable them to act with due skill and care. Continuing professional development (CPD) enables a professional auditor to develop and maintain the capabilities to perform competently within the professional environment.

There are currently 666 governmental auditors in Taiwan. All of them are either highly educated or richly experienced, or both, as they had first been required to have passed the National Civil Service examinations in accounting and auditing. Most of them are certified public accountants, engineering specialists, or certified internal auditors. In professions other than auditors, such as teachers, nurses or social workers, self-efficacy has been used to evaluate job satisfaction, job performance, career development, and health promoting behavior. The findings from these professions demonstrate the importance of self-efficacy for predicting and improving work performance. If an auditing quality is sensitive to ability, effort, and persistence, then efforts made to change self-efficacy and professional development (by changing beliefs, information, and knowledge) should improve performance. Although previous research in auditing accountability, mandate, function, procedures, practices and auditor independence, personality traits, job stress, organizational commitment and intention to leave have been discussed, the relationship between the self-efficacy, professional development and auditors’ audit quality has not yet been fully investigated.

**Fig. 1:**
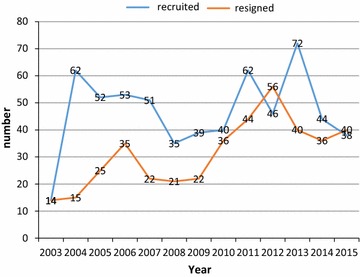
The dynamic changes of personnel between 2003 and 2015

In recent years, the Taiwan government has shifted its auditing focus from a more concern with legality and financial regulation to that of economic, efficient, and effective performance auditing, which is arguably more diverse and complicated. As creating and cultivating a developmental network is an arguably optimal approach to ensure continuous improvement in a rapidly changing work environment (such as globalization, etc.), one such strategy is to develop a mentoring system. High-quality mentoring is characterized by mutual learning, wherein partners can experience an increasing sense of work, knowledge, empowerment, and enthusiasm, as well as a desire for more connections. The auditors have large workloads. In 2012, they inspected approximately 8800 central and local governmental organizations, and audited the spending of regular and special budgets totaling US$601 billion. On average, each auditor was responsible for 13 organizations and US$902 million. Time constraints and workload pressure can reduce efforts toward knowledge sharing. Workload pressure may degrade both the extent and quality of knowledge sharing among members of an auditing team. Due to the stressful working environment, personnel loss is a key factor which cause instability of the structure in the audit organizations (Fig. 1). Such problem significantly lower the audit quality. In order to retain auditors, audit authorities must develop an enticing future outlook that emphasizes feedback and learning within the organization.

The purpose of this study is to advance the understanding of factors that may enhance auditors’ audit quality and to ultimately provide practical recommendations for the audit authorities. To evaluate whether audit quality can be improved by the professional development and self-efficacy of auditors, we conducted a large-scale survey of auditors working for Taiwan government. The results allowed us to propose a series of management recommendations to assist auditors in enhancing professional growth and increasing self-efficacy.

## Literature review

### Self-efficacy

Auditors do not work in isolation. It is crucial to understand how the people, tasks, and environment that auditors interact with influence auditor performance. Bandura (1977) proposed that individual behavior is a result achieved from interactions between environment and personal factors. Although self-efficacy is individual’s subjective view of one’s own ability, it profoundly influences personal actions, motivations, persistence, and therefore, the ultimate behaviors (Bandura 1991). Gist and Mitchell (1992) stated that self-efficacy is an important motivational construct. It influences individual choices, goals, emotional reactions, effort, coping, and persistence. Self-efficacy relates to individual task performance. Self-efficacy has a positive impact on performance because high self-efficacy enables the effective regulation of human behavior through a range of cognitive, motivational, and affective decisional processes (Bandura 1997).

Some of the determinants of self-efficacy are well-recognized, attributed causes (i.e., effort, ability, task difficulty). Bandura (1986) pointed out that persistence and level of effort mediated the relationship between self-efficacy and performance. Bandura (1989) stated that confidence resulted from successful execution has a positive impact on performance. Individuals which high self-efficacy approach difficult tasks as challenges to be mastered, rather than as threats to be avoided. These individuals set themselves challenging goals, maintain a strong commitment to these goals, and persist in their efforts in the case of a failure. Successful experience not only increases personal expectation on control and maturity of associated actions, but also provides source of self-efficacy for next challenge. Many studies have demonstrated that level of self-efficacy can predict work attitudes, job training, work performance, job satisfaction, educational development, and knowledge sharing (Randhawa 2004; Cabrera et al. 2006; Hsu et al. 2007; Hoy and Miskel 2001). Therefore, self-efficacy is widely perceived as one critical factor in determining how much effort and resources a person invests when confronting challenges.

Since self-efficacy is an important construct, it can increase energy, provide direction, and stimulate persistence (Porter et al. 1974). In fact, self-efficacy plays an important role for all professionals, including auditors. Hayati et al. (2014) stated that five job characteristics including skill variety, task identity, task significance, feedback and authority have play a critical role in growing work motivation. As auditing is a profession that provides services based on knowledge and experiences with human resource as the key element, the motivation and aptitude of an auditor to accomplish a goal is a strong advantage. Auditors with higher self-efficacy are more likely to continue investing in goal-achievement behaviour. Therefore, self-efficacy will influence behavior by affecting motivation and confidence to overcome difficulties and improve performance.

The strong positive relationship is the result of past performances influence on self-efficacy. Hoy and Miskel (2001) believed that the past work performance has a significant impact on the individual’s self-efficacy. The continuous success would definitely enhance individuals’ self-efficacy whereas the constant failures would create personal doubt and reduce personal self-efficacy. Since auditors accumulate knowledge and experience from clients to make professional judgments, auditing experience and professionalization can influence professional awareness. Moriarity (1979) found better performance by experienced auditors at bankruptcy prediction, which reflected superior ability of experienced auditors at auditing tasks. More specifically, perceptual self-efficacy is the basic element of initiative and the level of self-confidence necessary to accomplish a goal. People with high self-efficacy are more confident in their intellectual ability to allocate resources, control situations, and make critical decisions. Similarly, Cervone et al. (1991) observed that individuals with high self-efficacy learn more from feedback, respond more adaptively to decision environment, and overtime, are better able to translate their learning into performance. Charkhabi et al. (2013) found that when people with high self-efficacy encounter academic problems, they are less likely to give up and would try to find useful solutions to fix the problems. Therefore, to successfully resolve a challenge and complete an auditing task, the problem solver must draw upon experience, knowledge, and cognitive abilities. As specific knowledge is accumulated and more auditing skills are being developed, auditors become more likely to produce professional and comprehensive auditing reports.

### Professional development

Professional development is a learning process that can promote personal growth, improve auditing skills, revolutionize working procedures, and increase audit report quality. Due to many uncertainties of the audit process and unobservable characteristics of the results, audit specialization is proved to be associated with the capacity of error detection, procedure analysis, audit risk evaluation, and disclosure of internal deficiency (Stephens 2011). Knowledge is the primary input factor in producing an audit. The quality of the audit depends on the quality of auditor judgements during all stages of the audit, including risk assessment, internal control evaluation, testing, and review. Much research demonstrates the positive effects a good quality control and review processes on audit quality (Epps and Messier 2007; Bedard et al. 2008). Professional judgment determines audit procedures, and professionalization provides an advantage in client disputes. The maintenance of professional competence requires a continuing awareness and an understanding of relevant technical, professional and business developments. One of the strategies to advance a better performance is utilizing the modern technology. Technological advancements enable auditors on engagement teams to conduct electronic reviews of clients’ workpapers in their offices or from remote locations (Brazel et al. 2004). Audit software reduces the time required for workpaper preparation. Dodgson (1993) found that the value of knowledge can increase exponentially when it is networked, reused, and quickly integrated into business practices and processes. Such applications include decision support and expert systems, expert knowledge for specific problems, and point-to-point knowledge. Staff members can access industry best practices, studies, surveys, statistics, and expert knowledge for specific problems (Silvi 2002).

An auditor’s effort level needs to be tailored to each client within the structure of the basic audit methodology as applied by the audit team using their best judgement. Professional skepticism as well as auditor knowledge and expertise increase the quality of auditor judgements. Thus, the quality of the audit is based on auditor’s professional judgement. Auditors’ perceived goals of the audit and perceptions of how the audit authority values them influences auditors’ judgments. The resources needed for an audit depend on the personnel available for an engagement, the abilities and expertise of the audit team, and the audit technology and methodology being used. Consequently, assigning personnel with the appropriate levels of technical training and proficiency to audit engagements is required. Auditors can advance their personal development through continuous learning to increase their knowledge, open-mindedness, sensitivity to fraud detection, to set career goals, and to promote peer learning.

Auditors’ learning on the job and their choice of professional services jointly affect audit quality. The learning effect has a favorable impact on audit quality (Low 2004). While performing audits over time, auditors accumulate client-specific knowledge so that their posterior beliefs about clients are updated and become more precise. Bobek et al. (2012) pointed out that audit team communication, auditor–client negotiation strategy, and usefulness of prior auditing experience are significantly related to successful resolution of audit challenges. In addition, Bierstaker and Wright (2001) found that both ability and experience are determinants of performance on an ill-structured analytical review task and an ill-structured internal control auditing task. Ill-structured problems are routinely encountered in auditing. In order to solve an ill-structured problem, a problem solver must draw on experience, knowledge, and cognitive abilities. Vera-Muñoz et al. (2006) emphasized the importance to audit effectiveness of audit team members sharing knowledge and expertise with each other to affect a favorable audit outcome. Over time, auditors gain more client-specific knowledge, which is proportional to audit performance (Beck and Wu 2006). Auditor knowledge and expertise are also associated with superior performance in an audit (Nelson and Tan 2005). Also, experience affords opportunities to gain additional knowledge—which when combined with ability—positively affects performance (Libby and Luft 1993).

Industry specialization has become an element to not only provide audit quality but also maintain competitiveness (Miguel 2013). An organization can promote long-term efficacy and survival development through proper knowledge management. Knowledge is essential for maintaining competence. Similarly, knowledge is an enduring advantage that is constantly stimulated and accumulated to evaluate new experience and integrate information (Davenport and Prusak 1998). Due to the riskiness of audits and the idiosyncratic nature of audit engagements, Nelson (2009) pointed out that auditing requires various skills such as industry specialization or high level auditing. An auditor’s with industry expertise has been found to be positively related to the quality of audits. An auditor who is more knowledgeable in the audited industry has greater audit ability. Audit experience and professionalization provide positive influence to professional skepticism, which the audit professional judgment can be raised by professional skepticism and auditors’ knowledge and specialty. Auditor knowledge and expertise has a direct bearing on the audit quality. Auditors accumulate knowledge and experience from clients to make professional judgments. From the aspect of industry specialization and client industry characteristics, an auditor with strong knowledge in the audit industry is more capable to detect fraud and more likely to allocate resources to recruitment, training, technology, and audit techniques to improve audit service quality (Green 2008). In addition, if the audit authority does not provide enough information and knowledge of the particular industry, this industry would be less represented, thus, auditors cannot accumulate audit experience for this industry, leading to poor audit quality.

Minix (1987) considered professional development is the willingness to improve personal knowledge and skill through work. The value of knowledge is through sharing. Knowledge sharing can improve team performance and obtain best problem solving solution. Knowledge sharing can solve problems, avoid repeating mistakes and spread the adoption of best practices. It can enhance the effectiveness, efficiency and integrity of the audit process in formulating the most appropriate audit opinion. Stewart (1997) stated that transfer of knowledge and innovations create greater intelligent property for an organization. Workers’ knowledge and ability are the source of innovation and insight. The capital of innovation is accumulated by not only encouragement and investment from the organization but also personal creativity growth and development of workers. Huang (2009) stated that organization learning is how an organization accumulates knowledge and improves organization performance through workers’ skills to effectively integrate relevant risk experience and knowledge for developing strategies to deal with potential crisis. Hendriks (1999) proposed incentives of knowledge sharing are sense of accomplishment, responsibility, feeling appreciated, operational independence, promotion opportunity, and work challenge. Many studies have also suggested that organizations need to develop capacity to improve core procedures and continuous learning to maintain competitive advantage (Hall 2001; Jashapara 1993; Senge 1990; Johnson 2002). On the other hand, improper organization structure, poor sharing atmosphere, and fractionation would hinder knowledge sharing (Davenport and Prusak 1998).

Sadler (1988) proposed that the culture of an organization is a crucial factor affecting attitudes toward communication and communication processes and systems. Organizational culture represents the tacit norms, shared values, beliefs, and daily practices that shape the patterns and qualities of interactions between employees at different hierarchical levels. Senior management team may potentially mitigate audit challenges, and aid in successful resolution of challenges that arise. In general, a fair process builds trust and commitment and produces voluntary cooperation. Voluntary cooperation drives performance, thus leading people to go beyond the call of duty by sharing their knowledge and applying their creativity. In addition, mentoring can bring an array of important benefits at a reasonable cost. Anticipated outcomes of a mentoring program for individuals in a learning organization include acquiring, comprehending, and applying new knowledge in daily tasks; individually and collaboratively analyzing problems and proposing solutions; evaluating new technologies or strategies and determining their utility; and creating new business plans to improve organizational performance (Klinge 2015). Lange et al. (2015) suggested that for professional service workers, immediate work relevance of continuing professional development activities was the key determinant of the type of CPD activity rather than longer term career progression. Performance is used to refer to the individual’s ability to be creative, innovative, inspiring, and take on challenging tasks to achieve organizational goals for the greater good. Therefore, audit authorities must create an environment where achieving high quality is valued, nurtured, and rewarded. Such a requirement contributes to the profession’s objective of providing high-quality services to meet the needs of the public.

### Audit quality

DeAngelo (1981) defined audit quality as “the market-assessed joint probability that a given auditor will both (a) discover a breach in a client’s accounting system and (b) report the breach.” Based on this definition, audit quality can be broken down into two components: (1) the likelihood that an auditor discovers existing misstatements and (2) the likelihood that an auditor appropriately reacts to the discovery. The first component links to an auditor’s competence and degree of effort, while the latter relates to an auditor’s objectivity, professional skepticism and independence. In addition, the effect of audit quality should be determined according to the maturity of the execution conditions of all key factors that influence the mission performance of audit authorities. Knechel et al. (2013) stated that audit quality is conceived differently in different aspects. For the economic supervision, high audit quality means no major mistakes in the financial report. On the other hand, supervising management authority emphasizes high audit quality ought to meet professional standards. For auditors, high audit quality implies that the audit is accomplished according to the methodology or guideline defined by the audit authority. Whereas for audit authorities, high audit quality means that the audit report can withstand the challenge of court. As noted by social scientists, factors that can improve audit quality include (1) intensive training (Knechel et al. 2013), (2) audit specialization and execution of error detection, procedure analysis, audit risk evaluation, and internal control deficiency discovery (Stephens 2011), (3) the knowledge and skills to make professional decisions (Knechel 2010; Bobek et al. 2012), and (4) the professionalism of the auditors (Nagy 2012). Quality of people, processes, and business plans, those are vital for conducting an efficient and effective audit.

Wallman (1996) pointed out that audit quality is influenced by laws, regulations, economy, and culture. Carcello et al. (2002) indicated that audit quality is directly linked to the amount of audit work. Staffing and budget pressures continue to be a threat to audit quality. The time pressure also impact the quality of audit. The budget that did not include enough time for the engagement and increase the likelihood of engaging in reduced audit quality acts, happens often and causes the team to perform a lower quality audit in order to try to meet the budget. Therefore, audit quality would be determined by individual’s ability to make observation, manage information, and apply knowledge systematically and logically. Auditors should adjust work load or acquire assistance according to risk factors, ensuring auditors have sufficient time and resources to deal with difficult issues.

Auditor knowledge-acquisition activities affect audit quality. Auditors’ learning on the job has a favorable impact on audit quality. Auditors can enrich their knowledge accumulation by performing audit services. Lately, audit procedures have devote more emphasis in understanding client’s industry and business environment. Knechel et al. (2013) stated that audit quality is a carefully designed audit process that recruits talented employees to be properly motivated and trained to understand inherent uncertainty and adjust audit strategy to a unique client situation. The U.K.’s Financial Reporting Council (FRC) (2008) identified five drivers of audit quality: (1) the culture within an audit firm, (2) the skills and personal qualities of audit partners and staff, (3) the effectiveness of the audit process, (4) the reliability and usefulness of audit reporting, and (5) factors outside the control of auditors affecting audit quality. The National Audit Office of the Taiwan aims to evaluate the audit quality in the following five areas: leadership, personnel, auditing, clients, and continual improvement. The purpose of auditors’ recommendations is to eliminate the government deficiency. Based on these areas auditors are expected to provide independent insight and to be forward-looking, as well as offering advice to improve the efficiency of government authorities they serve.

## Methods

### Survey participants

As of December 2014, there were 666 auditors in Taiwan government. All auditors surveyed in this study are highly educated, experienced, or both, and required to pass the National Civil Service Examinations in accounting and auditing. Most of their job titles are auditors or senior auditors.

The participants surveyed in this study all are employees of either the Central or local government audit authorities, including the National Audit Office and its subsidiary Audit Divisions and Offices, the Education and Agriculture Audit Division, the Construction of Transportation and Communication Audit Division, the Six Municipality Audit Divisions, and the 15 county Audit Offices. The survey was conducted using the stratified random sampling method from April to June 2013.

### Questionnaire design

The survey questionnaire comprised two parts. The first part included the collecting of basic personal information, such as gender, age, education, years of auditing, division affiliation, and job title. The second part included questions about self-efficacy, professional development and audit quality, and was designed on the basis of principal component factors and varimax rotation factor analysis so as to ensure that the questions can cover every conceivable perspective.

All questionnaire items were measured using a 5-point Likert-type scale (1 = *strongly disagree* to 5 = *strongly agree*). Fourteen items were designed according to the self-efficacy scales designed by Schwarzer and Jerusalem (1995) and by Tai (2006). 23 items were based on factors related to professional development, including learning motivation, as addressed by Ames (1992); knowledge sharing, as addressed by Hendriks (1999); and organization culture, as addressed by Wallach (1983). 28 items were based on the auditing-related audit quality; thus, the reliability of each structure was ensured (Nunnally 1978; Bagozzi and Yi 1988). All of these attributes were developed from information in the literature review and personal interviews with auditing-related assistant auditor generals and senior auditors. A pilot test was conducted by interviewing 6 senior auditors from New Taipei Municipality Audit Divisions to assess the reliability of the self-efficacy, professional development, and audit quality attributes. Some wordings in the questionnaires were rephrased to clarify the questions after the pilot test. And the items listed on the final survey were examined carefully to avoid repeated questions. The collected data exhibited high internal consistency with the overall values of Cronbach’s α coefficient. Expert validity and Cronbach’s alpha coefficients were used to examine reliability and validity of the questionnaire. Cronbach’s alpha coefficients for self-efficacy, professional development, and audit quality were 0.906, 0.937, and 0.940 respectively (Table 1).

**Table 1:** The reliability of each structure

Variable	Factor name	Factor loading	Cronbach α	Overall Cronbach α
Self-efficacy	Profession and experience	0.598–0.734	0.807	0.906
	Confidence and effort	0.535–0.718	0.839	
	Trend and performance	0.613–0.785	0.729	
Professional development	Organizational culture	0.534–0.791	0.913	0.937
	Learning motivation	0.474–0.822	0.899	
	Training opportunity	0.462–0.839	0.863	
Audit quality	Leadership management	0.717–0.841	0.931	0.940
	Profession quality	0.484–0.773	0.919	
	Mission goal	0.507–0.697	0.779	
	Client value	0.564–0.793	0.838	

In the questionnaire, self-efficacy consists of three dimensions: profession and experience, confidence and effort, as well as trend and performance. Professional development acquisition has three dimensions: organizational culture, learning motivation, and training opportunity. Audit quality comprises four dimensions: leadership management, profession quality, mission goal and client value.

### Sampling accuracy evaluation

The audit authority of the Taiwan comprises 36 assistant auditor generals (5.4 %), 277 senior auditors (41.6 %), 77 senior inspectors (11.6 %), 231 auditors (34.7 %), and 45 inspectors (6.7 %). Because the number and titles of government auditors’ positions are regulated by the law concerned, the accuracy of sampling can be evaluated based on the job titles of the respondents to ensure that our sample represented a subpopulation of the overall audit authority. A Chi squared test with *p* > 0.05 indicates no significant difference between our sample structure and the matrix structure.

**Fig. 2:**
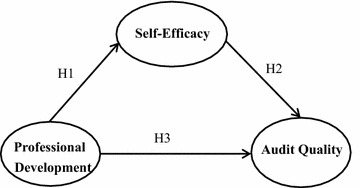
Research framework

The research framework of this study is illustrated in Fig. 2; the arrows indicate the direction of influence among the components. Among these variables, self-efficacy is widely thought to have a significantly positive correlation with performance in different fields. In this study, we investigated (a) whether auditors’ self-efficacy affects audit quality; (b) the major factors influencing the professional development of auditors; (c) the relationship between professional development and self-efficacy and (d) whether professional development through self-efficacy affects audit quality. After surveyed government audit staffs at various levels in Taiwan, we then used a survey research method to examine the hypothesized relationships among professional development, self-efficacy, and audit quality.

## Results

### Responses

Of the 339 questionnaires distributed to active auditors in Taiwan, 326 valid responses were returned, representing a very high response rate of 96.17 % and a sampling ratio of 48.95 %.

### Fair sample structure–matrix structure alignment

Our sample comprised 10 assistant auditor generals (3.1 %), 146 senior auditors (44.8 %), 31 senior inspectors (9.5 %), 118 auditors (36.2 %), and 21 inspectors (6.4 %). The survey results were not varied significantly according to job title (*p* > 0.05, Chi squared test), indicating that the survey sample structure is aligned with the matrix structure.

### Demographic variable adjustments

The survey subjects in this study can be categorized on the basis of the following factors (Table 2): (1) gender [women (52.1 %), men (47.9)]; (2) age [under 30 years (6.4 %), 31–40 years (40.5 %), 41–50 years (38.7 %), 51–60 years (10.8 %), 60 years above (3.6 %)]; (3) education level [master’s degree (51.2 %), bachelor’s degree (44.0 %)]; (4) auditing experience [more than 15 years (33.4 %), 11–15 years (17.0 %), 5–10 years (20.1 %), <5 years (29.5 %)], and (5) division affiliation [in local city or county Audit Offices (46.0 %), city Audit Divisions (30.1 %), National Audit Office (16.9 %), Audit Division on Education and Agriculture (7.0 %)].

**Table 2:** Sample demographics of the poll (n = 326)

Item	Percentage
*Sex*	
Male	47.9
Female	52.1
*Age*	
Under 30	6.4
31–40	40.5
41–50	38.7
51–60	10.8
60 above	3.6
*Job title*	
AAG	3.1
SA	44.8
SI	9.5
A	36.2
I	6.4
*Auditing experience*	
Under 5 years	29.5
5–10	20.1
11–15	17.0
15 above	33.4
*Education*	
Prof. school	4.8
Bachelor	44.0
Master	51.2
*Division*	
NAO	16.9
EA/TC	7.0
5 CAD	30.1
16 CAO	46.0

*AAG* assistant auditor general, *SA* senior auditor, *SI* senior inspector, *A* auditor, *I* Inspector, *NAO* National Audit Office, *EA/TC* Audit Division on Education and Agriculture, Audit Division on Construction of Transportation and Communication, *5CAD* 5 City Audit Division, *16 CAO* 16 County Audit Offices

### Current state of professional development

Results of the averages for all sections showed that “learning motivation” obtained the highest score (3.71), whereas “training opportunity” obtained the lowest score (3.34). In the “organizational culture” section, the “organization has established internal network to share knowledge and experience” received the most agreement (3.76); “organization frequently holds formal meeting to discuss and share knowledge” obtained lower score (3.15). In the “learning motivation” section, “my work provides opportunities to learn new knowledge” received the most agreement (3.94), followed by “I get a sense of accomplishment from work” (3.88) and “I learn from my and others experiences” (3.87). Furthermore, “I seek opportunities rather than wait for the occasion” (3.44) and “My work is stimulating and challenging” (3.43) obtained lower scores. Finally, for the “training opportunity” section, “the current work provides opportunities for me to learn and grow” received the most agreement (3.68); “organization provides training that responds to individual need” (3.15) and “organization trainings are sufficient to educate and improve the required skills” (3.11) obtained lower scores.

### Current status of self-efficacy

Results of the averages for all sections showed that “profession and experience” obtained the highest score (3.81), whereas “trend and performance” obtained the lowest score (3.53). In the “profession and experience” section, “I am professional” received the most agreement (3.87), followed by “I have practical audit experience” (3.84) and “my past achievements and experiences help me to increase my confidence level for success” (3.81). Moreover, “I can find several solutions when facing a difficulty” obtained lower score (3.73). In the “confidence and effort” section, “I can always solve a problem no matter how hard it is” received the most agreement (3.85); “I can easily stick to and achieve the goal” obtained lower score (3.10). Finally, for the “trend and performance” section, “I can plan and organize” received the most agreement (3.77), followed by “I have good oral communication skill” (3.46); “I am sensitive to the development of new technology and know how to apply it to my work” obtained lower score (3.36).

### Current state of audit quality

Results of the averages for all sections showed that “mission goal” obtained the highest score (3.85), whereas “leadership management” obtained the lowest score (3.47). In the “leadership management” section, “Supervisors inform my mistakes and advise me how to rectify them” received the most agreement (3.68), followed by “organization emphasizes team discipline” (3.64); “performance management system helps me understand what supervisors’ and organization’s expectation of me” (3.30) and “evaluation results reflect the performance” (3.29) obtained lower scores. In the “professional quality” section, “my work quality meets certain standards” received the most agreement (3.81); “I am visionary in auditing” obtained lower score (3.44). In the “mission goal” section, “I do my best to complete tasks” received the most agreement (3.98); “I clearly understand organizational goals and direction” obtained lower score (3.73). Finally, for the “client value” section, “I take responsibility to solve client problems” received the most agreement (3.62); “I give constructive advice to my client” obtained lower score (3.53).

### Difference analysis of survey subject attributes-influencing the professional development

Because of the background requirements and conservative organizational culture of the auditing profession, promotions and job performance are associated with seniority. Thus, the age of our respondents was highly correlated with seniority and job title (r = 0.734, 0.622, and 0.752, respectively). Therefore, we performed analysis of variance on various age groups on the basis of different years of experience and job titles (Table 3). The results revealed age to be a significant factor in *organizational culture* (*p* < 0.003). The results on age demonstrated that the average number was lowest in the age group of 31–40 years. Within *organizational culture*, the survey subjects from various age groups held different views on the following statements: “The organization established an internal network for sharing knowledge and experience,” “The organization emphasizes teamwork for reaching a consensus,” “The organization emphasizes innovative personal initiative,” “The organization encourages staff members to acquire knowledge to form new ideas and take action,” and “The organization frequently holds formal meetings to discuss and share knowledge.” Moreover, respondents with different years of auditing experience felt significantly different toward *organizational culture* (*p* < 0.010). The average number was lowest for those with 5–10 years of auditing experience. As mentioned, regarding the assessment of organizational culture, according to the number of years of auditing experience, respondents perceived the following statements differently: “The organization has established an internal network for sharing knowledge and experience,” “The organization frequently holds formal meetings to discuss and share knowledge,” “The organization emphasizes individual performance and growth,” and “The organization focuses on personal achievements.”

**Table 3:** Differentiated analysis on respondents’ attributes

	Age						Auditing experience					Job title					
	<30	31–40	41–50	51–60	>60	*p* value	<5 years	5–10	10–15	>15	*p*-value	A	I	SA	SI	AAG	*p*-value
*Professional development*																	
Organizational culture	3.58	3.39	3.50	3.76	3.89	0.003	3.44	3.34	3.56	3.63	0.010	3.37	3.66	3.50	3.65	4.27	0.000
Learning motivation	3.53	3.67	3.73	3.82	3.94	0.056	3.64	3.69	3.68	3.80	0.104	3.64	3.69	3.69	3.82	4.40	0.000
Training opportunity	3.36	3.27	3.33	3.48	3.77	0.100	3.28	3.22	3.43	3.41	0.180	3.27	3.38	3.29	3.51	4.15	0.001
*Self*-*efficacy*																	
Profession and experience	3.30	3.67	3.92	4.05	4.27	0.000	3.51	3.78	3.85	4.06	0.000	3.57	3.75	3.91	4.00	4.54	0.000
Confidence and effort	3.33	3.45	3.57	3.78	4.04	0.000	3.38	3.50	3.54	3.72	0.000	3.40	3.56	3.56	3.74	4.42	0.000
Trend and Performance	3.32	3.35	3.64	3.78	4.00	0.000	3.32	3.53	3.51	3.73	0.000	3.37	3.56	3.56	3.71	4.37	0.000
*Audit quality*																	
Leadership management	3.57	3.38	3.49	3.64	3.77	0.058	3.43	3.35	3.48	3.58	0.115	3.34	3.74	3.45	3.69	4.18	0.000
Profession quality	3.31	3.53	3.75	4.00	4.16	0.000	3.39	3.67	3.72	3.91	0.000	3.45	3.64	3.78	3.86	4.43	0.000
Mission goal	3.70	3.74	3.89	4.10	4.17	0.000	3.72	3.81	3.82	4.00	0.000	3.74	3.79	3.88	3.92	4.58	0.000
Client value	3.29	3.46	3.64	3.90	3.92	0.000	3.41	3.49	3.68	3.74	0.000	3.41	3.49	3.64	3.81	4.33	0.000

Regarding various job titles, the average number of assistant auditor generals was higher than that of senior auditors, senior inspectors, auditors, and inspectors. Assistant auditor generals, who are competent and possess vast practical experience, exhibited more advantages compared with auditors and inspectors, who in turn scored higher than did assistant auditors and assistant inspectors. Their abundant practical auditing experience and excellent performance history enable them to understand the profession and culture more in depth, and to advance professional development programs that inspire learning, stimulate knowledge sharing, and promote self-realization.

The survey results revealed that the lowest participant scores were from those in the age group of 31–40 years, with between 5 and 10 years of experience in *organizational culture*. Because the auditors in this age group were facing promotional pressure at this critical stage in their career, they wanted to believe that most problems could be solved through hard work. Employees hope that their organizations can focus on personal achievements.

### Professional development and self-efficacy on audit quality

Because the majority of the survey respondents were from two age groups [i.e., 31–40 years (40.5 %) and 41–50 years (38.7 %)], we further analyzed the effects of professional development and self-efficacy on the audit quality on the basis of gender, age, and auditing experience (Table 4). The *p* values indicated the degree to which each statement affected the audit quality. (a) The result from the male auditors between the ages of 31 and 40 indicated that *organizational culture, learning motivation*, as well as *confidence and effort* affected audit quality. (b) The female auditors of the same age group indicated that *organizational culture*, *training opportunities*, *confidence and effort*, and *trend and performance* affected the audit quality. (c) For auditors aged 41–50 with more than 15 years of auditing experience, the men indicated that *organizational culture*, *training opportunities*, and *profession and experience* affected the audit quality. (d) As for women in the same age group also with 15 years of experience indicated that *organizational culture*, *profession and experience*, as well as *trend and performance* affected the audit quality.

**Table 4:** The differentiated analysis of gender, age, and experience

Gender	Age				Auditing experience			
	31–40		41–50		5–10		>15	
	M	F	M	F	M	F	M	F
${\text{R}}_{\text{adj}}^{2}$	0.767	0.616	0.714	0.754	0.793	0.496	0.737	0.746
*Professional development*								
Organizational culture								
Mean	3.48	3.31	3.62	3.42	3.31	3.36	3.79	3.46
*p*-value	0.000	0.000	0.041	0.000	0.067	0.038	0.003	0.002
Learning motivation								
Mean	3.78	3.57	3.78	3.69	3.78	3.61	3.89	3.70
*p*-value	0.000	0.415	0.353	0.722	0.239	0.233	0.859	0.851
Training opportunity								
Mean	3.26	3.27	3.49	3.22	3.15	3.28	3.60	3.21
*p*-value	0.170	0.047	0.047	0.112	0.075	0.005	0.024	0.974
*Self*-*efficacy*								
Profession and experience								
Mean	3.74	3.62	3.88	3.95	3.79	3.78	4.10	4.00
*p*-value	0.800	0.355	0.046	0.001	0.145	0.107	0.016	0.008
Confidence and effort								
Mean	3.56	3.35	3.64	3.52	3.56	3.45	3.86	3.57
*p*-value	0.009	0.02	0.658	0.055	0.013	0.496	0.910	0.303
Trend and performance								
Mean	3.46	3.27	3.73	3.57	3.59	3.48	3.89	3.56
*p*-value	0.373	0.021	0.775	0.002	0.220	0.111	0.266	0.003

### Impact analysis of each factor

To analyze the impact of each factor on audit quality, we performed a regression analysis and obtained a model:

$${\text{y}} = .516 + .263{\text{x}}_{1} + .199{\text{x}}_{2} + .130{\text{x}}_{3} + .125{\text{x}}_{4} + .084{\text{x}}_{5} + .066{\text{x}}_{6.}$$

The results of regression analysis of professional development (organizational culture, learning motivation, and training opportunities), self-efficacy (profession and experience, confidence and effort, as well as trend and performance) on audit quality indicated that Table 5 (a) organizational culture, (b) profession and experience, (c) trend and performance, (d) confidence and effort, and (e) training opportunity significantly positively affected audit quality. The obtained F-ratio for the significance of multiple R was equal to 73.7. The square of multiple R (R^2^) was 0.732 suggesting that all the six predictors jointly accounted for 73 % of the total variance in audit quality. Among these factors, organizational culture, profession and experience, as well as trend and performance were the most crucial.

**Table 5:** Regression analysis on audit quality

Independent variables	Understand (*β*)	(*β*)	*T*-*value*	R^2^
Constant	0.516			
Organizational culture (x_1_)	0.263	0.368	8.730***	0.737
Profession and experience (x_2_)	0.199	0.231	5.436***	
Trend and performance (x_3_)	0.130	0.175	4.421***	
Confidence and effort (x_4_)	0.125	0.163	3.403**	
Training opportunity (x_5_)	0.084	0.132	3.324**	
Learning motivation (x_6_)	0.066	0.072	1.669	

** *p* < 0.01; *** *p* < 0.001, *F* value = 149.095, adjusted *R* ^2^ = 0.73

Because of the rapid development of science and technology, performance audits are now being associated with numerous fields, such as the policy sciences. A competent auditor is an expert on audit theory and has expertise in combining technological, managerial, and other relevant information on technology concepts to design a more efficient work process, thereby reducing workloads and allowing more time for training.

### Regression analysis on professional development, self-efficacy, and audit quality

Table 6 lists the linear effect of the independent variables on the dependent variables. Self-efficacy had a significantly positive effect on audit quality (*β* = 0.735, *p* < 0.001), and the explanatory power was 54.0 %. A detailed analysis of each aspect of self-efficacy revealed that *profession and experience*, *confidence and effort*, as well as *trend and performance* had a significantly positive impact on *profession quality* for audit quality (*β* = 0.766, 0.725, and 0.707, *p* < 0.001). Furthermore, *profession and experience*, *confidence and effort*, as well as *trend and performance* had a significantly positive effect on *mission goal* for audit quality (*β* = 0.553, 0.592, and 0.556, respectively, *p* < 0.001).

**Table 6:** Regression analysis on audit quality

Variable name	Audit quality			
	Leadership management	Profession quality	Mission goal	Client value
*Professional development*				
Organizational culture				
(*β*)	0.750	0.389	0.499	0.334
*t*-value	20.402***	7.606***	10.351***	6.387***
Learning motivation				
(*β*)	0.500	0.562	0.594	0.471
*t*-value	10.397***	12.219***	13.296***	9.619***
Training opportunity				
Mean	0.608	0.378	0.404	0.324
*t*-value	13.792***	7.347***	7.951***	6.166***
*Self*-*efficacy*				
Profession and experience				
(*β*)	0.241	0.766	0.553	0.504
*t*-value	4.473***	21.440***	11.941***	10.516***
Confidence and effort				
(*β*)	0.418	0.725	0.592	0.504
*t*-value	8.291***	18.941***	13.206***	10.510***
Trend and performance				
(*β*)	0.298	0.707	0.556	0.462
*t*-value	5.620***	17.985***	12.046***	9.364***

*** *p* < 0.001

Overall, professional development had a significantly positive impact on audit quality (*β* = 0.766, *p* < 0.001), and the explanatory power was 58.7 %. A detailed analysis of each aspect revealed that *organizational culture*, *learning motivation*, and *training opportunities* had a significantly positive effect on *leadership management* for audit quality (*β* = 0.750, 0.500, and 0.608, respectively, *p* < 0.001). Moreover, *learning motivation* significantly affected *profession quality* for audit quality (*β* = 0.562, *p* < 0.001). *Organizational culture* and *learning motivation* were found to have a significantly positive effect on *mission goal* for audit quality (*β* = 0.499 and 0.594, respectively, *p* < 0.001).

### Discussion on mediating effects

For this study, we used the variance inflation factor (VIF) to test the collinearity. Because the VIF value was 1.476 (<10), the data set was not collinear. *Professional development* (*β* = 0.568, *p* < 0.001) positively affected *self*-*efficacy*. Moreover, *professional development* (*β* = 0.766, *p* < 0.001) and *self*-*efficacy* (*β* = 0.735, *p* < 0.001) had a significantly positive effect on audit quality. In further considering the mediator, we determined that for *professional development* (*β* = 0.515, *p* < 0.001), self-efficacy remained significant (*β* = 0.443, *p* < 0.001; Table 7). These results revealed that self-efficacy exhibits a partial mediating effect, but without collinearity.

**Table 7:** Mediating effect analysis

Dependent variables	Independent variables	(*β*)	*t*-*value*	R^2^	F-*value*	VIF
Self-efficacy	Professional development	0.568	12.416	0.322	154.156	1.000
Audit quality	Self-efficacy	0.735	19.511	0.540	380.677	1.000
Audit quality	Professional development	0.766	21.469	0.587	460.911	1.000
Audit quality	Self-efficacy	0.443	12.372	0.720	415.156	1.476
	Professional development	0.515	14.397			

Self-efficacy has been shown to have positive impact on performance management in various fields. This study explored if the same relationship exhibits in audit profession in which the demand on audit quality are ever increasing. By the wave of promoting performance audit, auditors are under increasing pressure to raise performance. The study showed that professional development has partial mediating effect on audit quality.

## Discussion

Audits optimize government function by evaluating the legitimacy, economy, efficiency, and effectiveness of how administrative branches utilize resources. For example, audit authorities have revealed that the Taiwan government previously constructed so-called “mosquito buildings” (idle buildings “used only by mosquitoes”) to create an illusion of public construction achievement and opportunities for kickbacks. Such construction squandered and unevenly distributed government funds. The number of mosquito buildings reached of 163, but an effective auditing has reduced this figure to nine. Auditing reports can effectively provide opinion and suggestions to further improve the performance of the executive branches. The key to quality auditing is to review policies from an international perspective so as to provide insight, predictions, and warnings for comparison.

The purpose of this study was to examine the relationships between professional development, self-efficacy, and audit quality. The ordinary least squares (OLS) to perform multiple regression analysis has been utilized to calculate the coefficient estimates. Self-efficacy (*β* = 0.568, *p* < 0.001) is found to have been influenced by professional development, and thus supporting H1 as described in (Fig. 2). As predicted in H2, self-efficacy has influenced audit quality (*β* = 0.735, *p* < 0.001). Finally, audit quality has been found to have been influenced by professional development (*β* = 0.766, *p* < 0.001), and thus supporting H3. Self-efficacy and audit quality have found to have a positive correlation. High self-efficacy typically leads to higher audit quality, and self-efficacy has immense effects on an individual’s motivation, effort, persistence and performance. Professional development and audit quality have found to have a positive correlation. High profession growth typically leads to higher audit quality. Therefore, self-efficacy and professional development affects audit quality.

We have found that most respondents have believed that there are numerous opportunities for personal growth in their organizations and that their jobs have provided opportunities for future development. This is because auditors are routinely assigned tasks that vary in complexity and industry. Job enrichment provides a sense of control over one’s work environment and motivates people to exercise their full potential, thus presenting more opportunities for employee success. However, they have also believed that audit authorities have not provided enough knowledge to enable them to share auditing experiences and educational training. Auditors must share knowledge and expertise on industry-specific trends with members of the audit team as well as their accounting, auditing, and regulatory concerns that may influence the performance and outcome of an audit. In addition, audit authorities must create, integrate, share, and use knowledge regarding their clients’ control activities and corporate governance. Implementing these knowledge-based activities effectively is increasingly critical for audit authorities for maintaining their competitive advantage, including gaining tangible benefits regarding time and cost reductions. Thus, sharing knowledge can aid audit authorities in leveraging the skills, knowledge, and optimal practices of their professional staff members. More training and recruitment of talented employees would enhance audit processes which in turn would have a favorable impact on audit outcomes.

We have also found that auditors generally have a positive attitude toward their professional abilities and experience, confirming the view that experience can increase self-efficacy. However, auditors were found to be less confident in communication skills and remaining abreast of technological developments, indicating that audit methodologies, work procedures, and communication with clients can be improved further. Working in a diversified environment, auditors must be administrative and management experts as well as internal-control designers for (a) adhering to audit principles and learning advanced auditing methods, (b) nurturing a sense of innovation for developing novel audit processes and methods, and (c) creating new audit-operating mechanisms. Audit quality reflects a carefully designed audit process that involves recruiting talented employees to be properly motivated and trained, thus enabling them to understand inherent uncertainties and adjust audit strategies to accommodate unique client conditions. Being efficiently and effectively requires continual education and training on mentor and knowledge sharing. The appropriate use of IT assets results in organizational innovations and facilitates redesigning business processes, and favorable competitive dynamics generate improvements in organizational performance because of such organizational innovations. Therefore, increasing the value of the audit profession in a complex audit environment necessitates constantly adapting to new types of technology and updating auditing concepts. Past work performance significantly affects an individual’s self-efficacy and that continual success indubitably enhances an individual’s self-efficacy, whereas constant failure creates personal doubt and reduces personal self-efficacy. Accordingly, the proposed methods for increasing the self-efficacy of auditors include the following: (a) successful experience from past assignments; (b) self-confidence in one’s potential for achieving goals; and (c) understanding that a performance audit is not a difficult task, but a favorable success model. Therefore, audit tasks should be rotated among staff members for enabling effective cross training. In this manner, staff members would become more versatile and strengthened by learning from one another, and auditors’ self-efficacy could thus be increased for maintaining high audit sensitivity.

The results of the survey revealed that auditors were typically satisfied with how they plan, execute, and accomplish tasks. Auditors usually followed standard procedures for accomplishing tasks. However, the audit field assignment was executed on the basis of task units. Because the knowledge and skills of auditing are complex, audit quality and performance rely on the specialization and audit environment. The auditing process usually concludes with a report, which is a compilation of reports from each member of the field task unit. Thus, teamwork affects the quality of audit reporting and requires that team leaders guide every member. Leaders should recognize that developing cooperative relationships among team members promotes team effectiveness. Therefore, teamwork has been proven to be a significant factor affecting the quality of an audit report. By contrast, an effective audit recommendation should be based on evidence that practically resolves issues in accordance with regulations. When auditors lack on-the-job training, they are incapable of issuing a fair judgment and thus cannot deliver a report, which would otherwise present opinions on how to effectively use a budget. Performance management can be improved by developing visionary thinking and providing constructive recommendations in an audit report. An audit report can provide information and useful references for further improving the performance of executive branches, thus preventing redundant “mosquito” museums, harbors, and facilities from being built.

Auditing experience and professionalization positively influenced professional skepticism, which, in addition to an auditor’s knowledge and skillset, can improve professional judgment. Experienced baby boomers are rapidly nearing retirement age, and their accumulated wisdom and expertise could soon be inaccessible. Shrivastava and Purang (2011) indicated that feedback was effective in the presence of a strong link between performance improvement and valued outcomes. Brown and Duguid (2000) found that the loss of professional autonomy associated with structured audit approaches increased the turnover rate among senior audit staff members and, by extension, resulted in the loss of knowledge possessed by exiting personnel. Leaders should create an open culture that is conducive to mentoring, where people learn from one another through a wide variety of formal and informal relationships at an enterprise level. Thus, everyone can reap the benefits of mentoring. Specifically, mentoring others or sharing knowledge can improve the efficiency and effectiveness of audit procedures.

The survey results revealed that the lowest participant scores were from those in the age group of 31–40 years, with between 5 and 10 years of experience in *organizational culture*, *training opportunit*ies, and *leadership management*. Because the auditors in this age group were facing promotional pressure at this critical stage in their career, they wanted to believe that most problems could be solved through hard work. Employees hope that their organizations can focus on personal achievements. They also wish to participate in official or unofficial meetings to discuss and share knowledge, thus enabling them to develop and grow on a broad scale. Thus, knowledge sharing can help audit authorities leverage the skills, knowledge, and optimal practices of their professional staff members. Auditors must share with members of the audit team their knowledge and expertise on industry-specific trends as well as accounting, auditing, and regulatory concerns that may influence the performance and outcome of an audit. In any organization, the highest performing individual is typically recognized. Performance evaluations can enable workers to gain an improved understanding of their work, performance, and even themselves. Moreover, they can improve mutual understanding between supervisors and workers, thereby inducing motivation. Furthermore, as a foundation for promotions and salary adjustments, performance management can be useful for “training and molding” as a method of instilling team spirit and a sense of unity. Thus, timely rewards that are provided to employees whose personal efforts yield high-quality audit can instill team spirit and a sense of unity.

Both male and female auditors in the age group of 31–40 years indicated that *organizational culture* as well as *confidence and effort* affected the audit quality. Because the auditors in this age group were facing promotional pressure at a critical stage in their career, they focused on personal achievements and knowledge sharing. When input effort is correlated with the performance outcome, most problems can be resolved with hard work. On the basis of past success, self-efficacy increases. By focusing on work, they can develop and experience a sense of fulfillment. As for the auditors in the age group of 41–50 with more than 15 years of experience indicated that *organizational culture* as well as *profession and experience* affected the audit quality. Abdolmohammadi and Wright (1987) reported that for unstructured tasks, the complexity of a task is most influenced by the experience of a worker. Bierstaker and Wright (2001) found that auditors’ experience significantly influenced their ability to solve practical problems. Experience had a positive impact on audit decision. Specifically, the male auditors in this age group with more than 15 years of experience indicated that *training opportunit*ies were more crucial. Their female counterparts indicated that *trend and performance* were more crucial because advances in technology can improve work efficiency, thereby increasing the time available for training, which enhances the audit quality.

We found that *profession quality* exerted a significantly positive effect on *profession and experience*, *confidence and effort*, as well as *trend and performance*, *leadership management* exerted a significantly positive effect on *organizational culture*, *learning motivation*, and *training opportunities*, indicating that an effective learning environment and adequate resources can enhance the potential of auditors. Learning from an audit system and from the experience of those from other countries, auditors in the Taiwan government have been advancing. Within an audit authority, an organization encourages team, model, and target learning to increase auditors’ professional growth. In utilizing information technology, organizational knowledge can be shared faster and easier. By sharing auditing experience, auditors can enhance their preparation by studying the relevant information of each stage before an audit. The audit would become more effective as a result of a more detailed and improved strategic inspection. Knowledge sharing and learning enhance audit value and facilitate the sustainment of high audit sensitivity, thereby enabling the audit report to provide more efficient and cost-effective recommendations for improving governmental performance.

## Conclusion

After having surveyed nearly 50 % of all the governmental auditors in Taiwan, we have found that experience to be one most significant factor in their auditing efficacy. Auditing experience and professionalization positively influenced professional awareness, which, with the knowledge and specialty of an auditor, can improve professional judgment. Audit authorities benefit from the implicit knowledge of their employees; people are the most crucial information carriers and the most abundant assets in governmental audit authorities. Therefore, with increasing age, experience, and position, government auditors become more equipped to identify critical errors, process analyses, evaluate audit risks, disclose internal-control mistakes, authenticate complex evidence charts, and issue professional judgments.

Auditors in the age group of 41–50 with more than 15 years of experience indicated that organizational culture as well as profession and experience had a significantly positive impact on leadership management, profession quality, mission goal, and client value in audit quality. However, senior auditors have been observed to have responded to workload pressure by expending little or no time in providing feedback to audit staff members under their charge. Also, auditor turnover has reduced knowledge sharing in an audit. On the basis of these results, we recommend that the Taiwan government improve its current working environment and enhance job training on mentoring. To foster knowledge sharing, recruitment and selection should favor people who are open to learning and using novel concepts and practices. Audit authorities should include specific guidance in their recruiting policies that will aid recruiters in identifying candidates who exhibit individual-level traits consistent with the organization’s goal and values that are commonly associated with the ability to work effectively in teams and share knowledge. Simultaneously, audit authorities can begin using technology to help people forge new relationships across traditional boundaries in order to expand learning networks. Making use of peer coaching, mentoring circles, and learning partners can provide favorable opportunities for individuals to build their own developmental networks. To enhance knowledge sharing between preparers and reviewers in the work-paper review process, training should be tailored to the specific needs of different ranks of auditors. In addition, audit authorities should encourage knowledge sharing, cross-training, and strategic job rotation among different generations to integrate baby boomers’ experience with the creativity of Millennials. Such practice can result in groundbreaking innovations.

According to examples from other countries, the National Audit Office of Taiwan evaluates audit quality in five areas: leadership, personnel, auditing, clients, and continual improvement. To improve communication among audited units, audit authorities should understand that reprimanding people or challenging policies is not the main purpose of a performance audit. Auditors should focus on the outcomes of a policy to obtain insightful results and recommendations for governing without interfering with the executive branch. Therefore, audit authorities positively affect society by increasing the economic value of audited units. Because the main aim of a performance audit is to improve government performance, audit authorities should strive to develop collaborative partnerships with the executive and legislative branches to aid in enhancing governance. Leaders should recognize that developing cooperative goals among team members is essential for reinforcing these values and ensuring the credibility. Our study showed that the evaluation, performance, and value of outputs and outcomes evidently rely on auditors’ self-efficacy as well as profession and experience. Auditors should maintain a strong and professional relationship with audited units, enabling them to appropriately communicate audit results, thereby effectively improving governing performance. Thus, the key to quality audit involves inspecting policies from an international perspective to provide insight, predictions, and warnings. According to the mission statement of the American Accounting Association, the auditing profession should “foster excellence in the teaching, research, and practice of auditing and assurance services.” Accordingly, the core competencies of auditors are as follows: (a) communication ability and leadership for inspiring people to achieve common goals; (b) the ability to conduct comprehensive financial analyses, provide insight, and offer constructive recommendations; and (c) an awareness of new technological trends and the ability to apply advanced technology to increase client and employee value. Audit authorities should operate on the basis of public governance perspectives and focus on regulatory and performance auditing to ensure that the executive branch utilizes funds legally, enabling it to economically and efficiently achieve goals.

Overall, the survey findings indicated that self-efficacy and professional development significantly influenced the audit quality. Employers should focus on improving employee self-efficacy for enhancing both individual and organizational performance. When they have assisted each other in achieving their tasks and goals, they would have felt the needed individual support being fully vindicated. Employee self-efficacy can be vicariously enhanced through counseling, job enrichment, proper guidance training, development programs, challenges, and autonomous jobs and rewards. In addition to establishing an internal network, organizations should hold frequent formal meetings to discuss and share knowledge and experience. They should also provide a knowledge-sharing platform and educational training to improve efficiency, thereby increasing the available training to enhance audit quality. When employees believe that they are advancing, their work is meaningful, and their working environment is conducive to develop, their teams are well-structured, and they have sufficient resources, they are most likely motivated to receive more training for better development and efficient execution. Supporting the professional growth of staff members will definitely improve their task performance and personal satisfaction.

To avoid discouraging auditors from responding to our survey, we had not included several performance variables such as recommendations and balances in audit reports, amounts returned to the national treasury, and the number of staff members who had been reprimanded. And future studies can include these variables for comparison. Arguably, audit quality is difficult to define and quantify, and we recommend future research using alternative measures of audit quality to validate and elaborate on our findings. Several factors examined such as professional development and self-efficacy in this study likely affect audit quality directly, whereas others are more likely to mediate or moderate audit quality. We will encourage future research that empirically examine direct, moderating, or mediating effects on audit quality so as to obtain a more comprehensive picture of good audit quality with respect to the area covered in this study.
